# Effects of Repeated Use and Sterilization on the Wear of Zirconia Implant Drills: A SEM‐Based Analysis

**DOI:** 10.1002/cre2.70088

**Published:** 2025-02-12

**Authors:** Vasilios Alevizakos, Richard Mosch, Ann‐Christin Platte, Constantin von See

**Affiliations:** ^1^ Research Center for Digital Technologies in Dentistry and CAD/CAM Danube Private University Krems an der Donau Austria

**Keywords:** dental implant, drill wear, sterilization, zirconium oxide

## Abstract

**Objectives:**

This study evaluated the effects of repeated use and sterilization on the wear and cutting‐edge integrity of zirconia implant drills.

**Materials and Methods:**

Sixty zirconium dioxide drills (Z‐Systems AG) with diameters of 2.3, 3.75, and 4.25 mm were tested. Drilling was performed in porcine mandibular bone under standardized conditions: 800 rpm, 50 Ncm torque, and 20 N axial pressure. Drills were divided into two groups: Group 1 (sterilized but unused) and Group 2 (30 drilling cycles with reprocessing). Wear was assessed using scanning electron microscopy (SEM) at 1000x magnification, applying a three‐grade scoring system. Statistical analysis was performed using the Mann–Whitney U test (*p* < 0.05).

**Results:**

Drills subjected to 30 cycles showed significantly higher wear grades (Grade 2–3) compared to unused drills (Grade 0–1) (*p* < 0.001). The mean wear grades increased from 0.3 to 2.6 for 2.3 mm, from 0.4 to 2.7 for 3.75 mm, and from 0.2 to 2.7 for 4.25 mm drills. Effect sizes (*r* = 0.88–0.90) confirmed a strong relationship between repeated use and wear.

**Conclusions:**

Zirconia drills show significant wear after 30 cycles. Although they offer potential as an alternative to steel drills, further research is needed to optimize cost‐effectiveness and clinical durability.

**Clinical Trial Registration:**

Not applicable.

## 1 Introduction

Dental implantation has become a routine and effective procedure in modern dentistry, offering solutions for single‐tooth replacements as well as fixed or removable multi‐unit restorations. The success of these procedures is influenced by a range of factors, with implant bed preparation being a critical determinant of long‐term outcomes (Stacchi et al. 2023).

The survival rate of dental implants is governed by an interplay of patient‐specific factors, clinical expertise, and material selection. Titanium and its alloys, as well as medical steel, have traditionally been the materials of choice for implant drills and implants due to their biocompatibility and mechanical properties (Guo et al. 2021). However, the search for alternatives to address limitations such as esthetic concerns, disruptions in oral homeostasis, microbiome alterations, and long‐term survival rates has directed attention toward ceramic materials (Lorusso et al. 2020).

Dental ceramics encompass a variety of types, including silicate ceramics, oxide ceramics, and non‐oxide ceramics, each with unique physical and mechanical properties. Lithium disilicate, a prominent silicate ceramic, is widely used for anterior restorations due to its translucency and sufficient flexural strength (Giordano Ii 2022). However, its application in posterior regions remains contentious due to the higher mechanical demands in these areas (Špehar and Jakovac 2024). In contrast, zirconium dioxide, an oxide ceramic, has superior flexural strength and durability, making it a preferred material for both tooth‐ and implant‐supported prosthetics (Alenezi and Aloqayli 2023).

Although titanium implants continue to dominate the market, zirconium implants are garnering increasing interest, though clinical evidence supporting their use is still evolving. Similarly, zirconia drills have been introduced as alternatives to traditional drills, and yet, their thermal conductivity, brittleness, and susceptibility to abrasion during implant bed preparation raise concerns (Cahill et al. 1991; Padhye et al. 2023).

From a clinical perspective, surgical experience, procedure duration, implant placement timing, sterilization protocols, and implant stability are pivotal to achieving successful outcomes. A key challenge during implant bed preparation is minimizing heat generation to avoid thermal damage, which could impede osseointegration. Frictional heat generation depends on factors such as bone density, drill design, speed, and sharpness (Jain et al. 2024; Jang et al. 2023; Aquilanti et al. 2023). Ensuring sharp cutting edges and the use of proper drilling techniques are essential to mitigating thermal damage and preserving bone tissue integrity (Eriksson and Albrektsson 1984).

Additionally, repeated use and sterilization of implant drills may degrade their cutting efficiency, potentially compromising surgical outcomes (Gehrke et al. 2018). This study seeks to evaluate the impact of multiple drilling cycles and sterilization processes on the integrity of zirconia drill cutting edges using scanning electron microscopy. It hypothesizes that repeated use and sterilization will significantly influence the cutting performance and wear patterns of zirconia drills.

## 2 Hypotheses

Repeated sterilization cycles and multiple uses significantly impact the cutting‐edge integrity and wear patterns of zirconia implant drills.

## 3 Materials and Methods

### 3.1 Study Design and Materials

**Figure 1:**
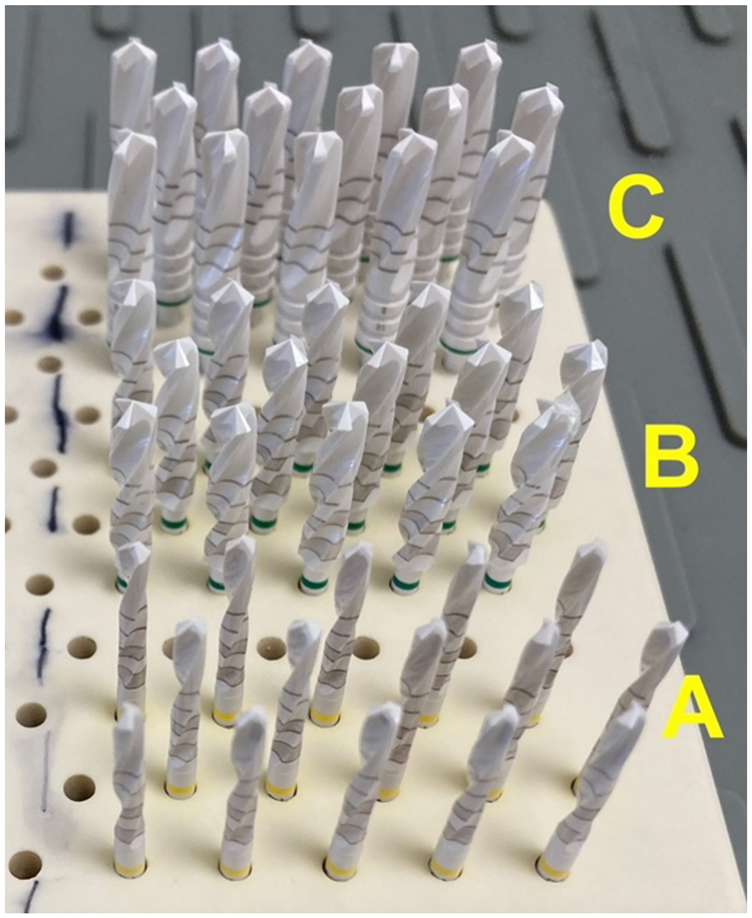
Zirconia implant drills from Z‐Systems. (A) implant drills with 2.3 mm diameter, (B) implant drills with 3.75 mm diameter, and (C0 implant drills with 4.25 mm diameter.

This study investigated the performance and wear characteristics of 60 twist drills manufactured by Z‐Systems AG (Oensingen, Switzerland), composed of zirconium dioxide. The drills, each with a total working length of 16 mm, were categorized into three diameters: 2.3 mm (*n* = 20), 3.75 mm (*n* = 20), and 4.25 mm (*n* = 20) (Figure 1).

### 3.2 Substrate Preparation

Fresh porcine jaws were used as the experimental substrate. Surrounding tissues and periosteum were meticulously removed to facilitate unobstructed chip evacuation during drilling. Drilling was conducted in the angulus mandibulae (jaw angle) region to ensure uniformity of the bone substrate.

### 3.3 Experimental Setup

**Figure 2:**
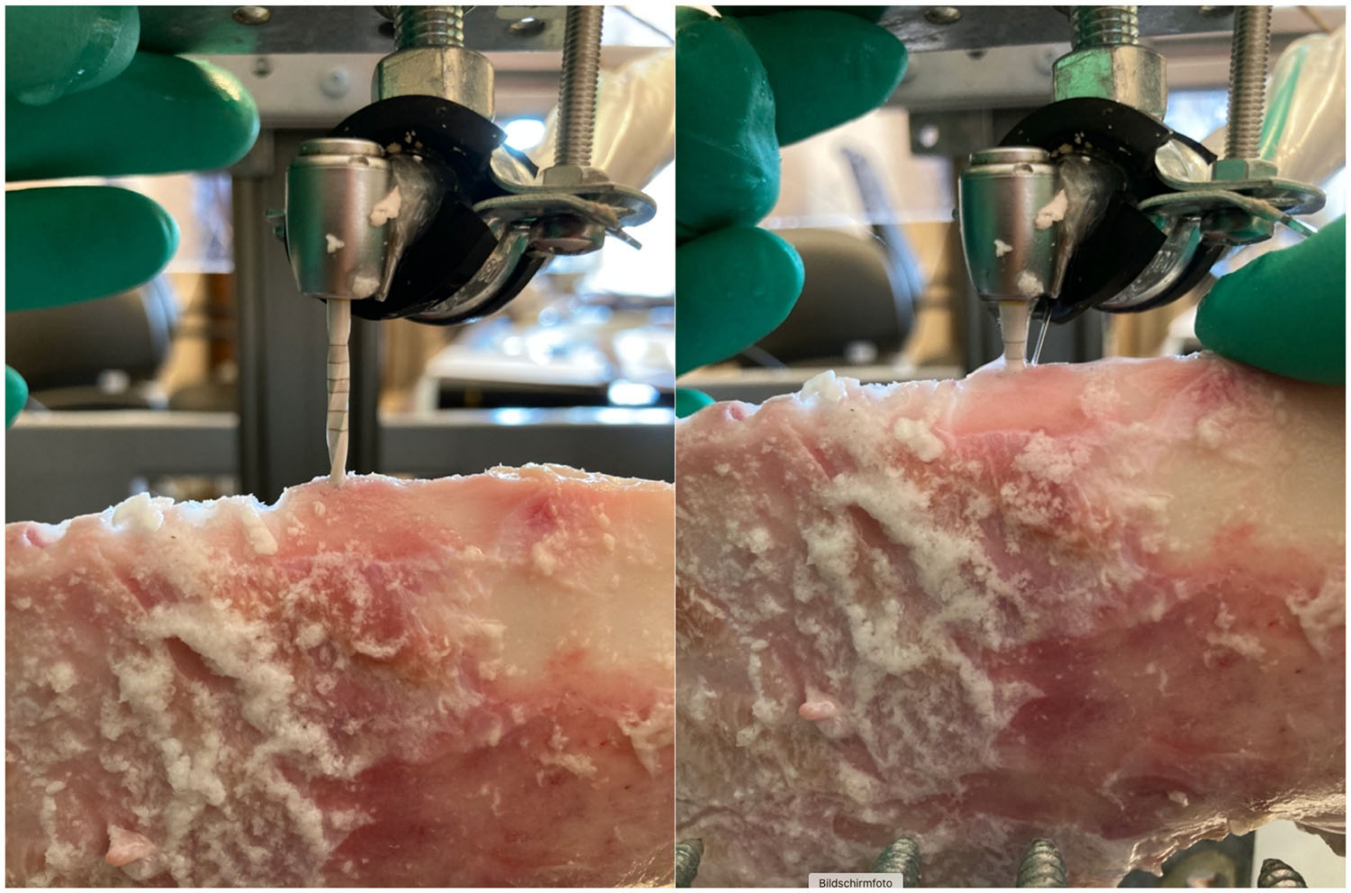
The left image shows the starting point of the measurement (the drill tip touches the bone surface). The right image shows the end point of the measurement (the drilling depth of 13 mm has been reached).

A custom‐designed apparatus comprising two interlocking frames (horizontal and vertical) was used to securely position the porcine jaws while enabling controlled drilling motions. A guide rail supported the test carriage to ensure perpendicular drilling into the substrate. Inductive proximity sensors integrated into the setup continuously monitored drilling depth and emitted an acoustic signal upon reaching the pre‐determined depth of 13 mm (Figure 2).

### 3.4 Drilling Protocol

Drilling was performed using a surgical contra‐angle handpiece (WI‐75 E/KM, W&H, Bürmoos, Austria) mounted on a guide rail for stability and control. The drilling parameters were standardized using the W&H Elcomed 100 Surgical Console System (Nobel Biocare, Yorba Linda, CA), with a drill speed of 800 rpm and torque set at 50 Ncm. External irrigation with a physiological solution was maintained at a constant flow rate of 200 mL/min to cool the drill and optimize performance. The contact pressure was set at 20 N to ensure consistency across all procedures.

### 3.5 Drill Reprocessing

The 60 drills were divided into two groups:

**Group 1:** Sterilized but unused drills.

**Group 2:** Drills subjected to 30 drilling cycles.

After each drilling session, bone chips were cleaned from the drills with water. The reprocessing protocol for Group 2 drills included machine cleaning, disinfection in a 2% disinfectant solution (ID 213 Instrument Disinfection, Dürr Dental SE, Bietigheim‐Bissingen, Germany) for 3 min in an ultrasonic bath (Elmasonic S 130H, Elma Schmidbauer GmbH, Singen, Germany), and sterilization in a vacuum autoclave (Universalprogramm, Vacuklav 40 B+, Melag, Berlin, Germany) according to the manufacturer's instructions. This disinfection protocol was applied consistently for all drills, ensuring standardization across both groups.

### 3.6 Wear Assessment

Post‐reprocessing, all drills were subjected to scanning electron microscopy (SEM) analysis at the Linz Institute of Technology to evaluate wear and deformation. The TM‐1000 microscope (Hitachi, Tokyo, Japan) was used at 1000x magnification to capture high‐resolution images of the cutting surfaces. Each drill was examined before and after the designated number of drilling cycles to assess alterations in geometry and structural integrity.

A three‐grade scoring system was used for qualitative assessment:

**Grade 0:** No discernible changes from the initial condition.

**Grade 1:** Visible alterations with intact, unchipped cutting lines.

**Grade 2:** Visible alterations with chipping along the cutting line.

**Figure 3:**
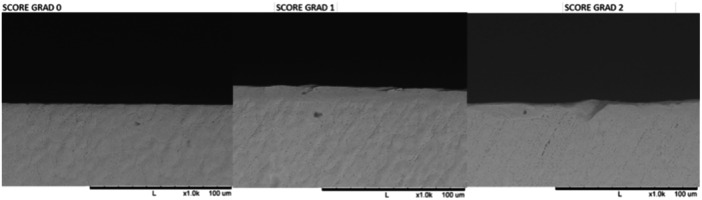
Three‐grade scoring system to quantify deviations in drill geometry and integrity: Grade 0: Signifying no discernible changes relative to the initial condition. Grade 1: Denoting visible alterations, with the cutting line appearing straight and devoid of chipping. Grade 2: Indicating visible changes with evidence of chipping along the cutting line.

**Figure 4:**
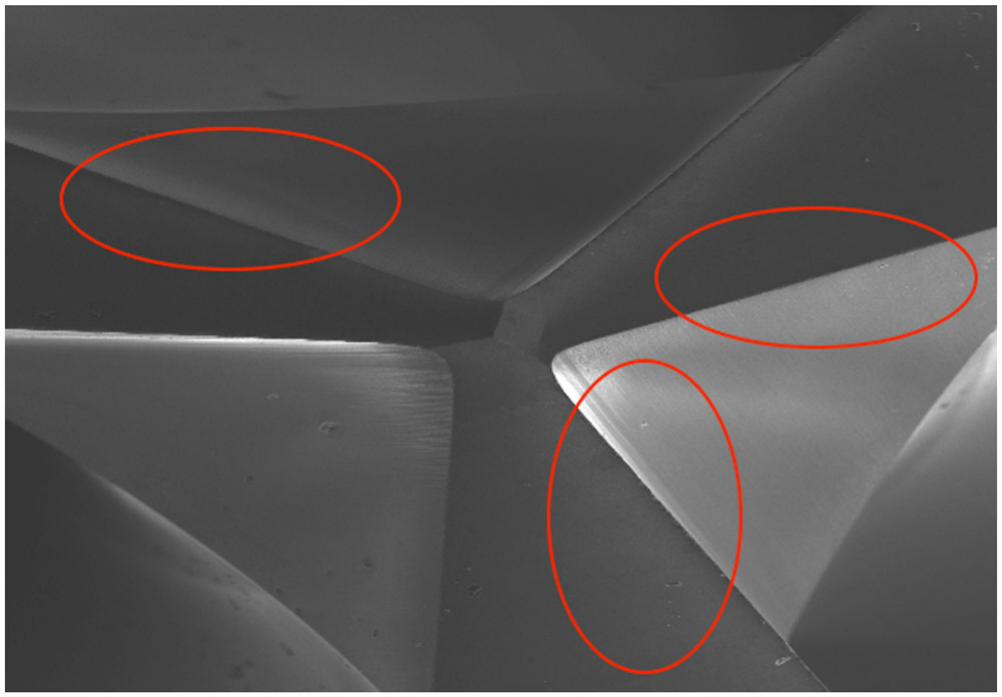
The investigated area, annotated within a red frame, provided a visual reference for assessing wear and deformation characteristics.

Figures 3 and 4 provide visual references for assessing wear patterns.

### 3.7 Statistical Analysis

Data from the SEM examination were analyzed using IBM SPSS Statistics 30.0.0. (Armonk, NY, USA). The Mann–Whitney U test was used to compare the mean values of the drill wear scores. Statistical significance was set at *p* < 0.05.

## 4 Results

**Figure 5:**
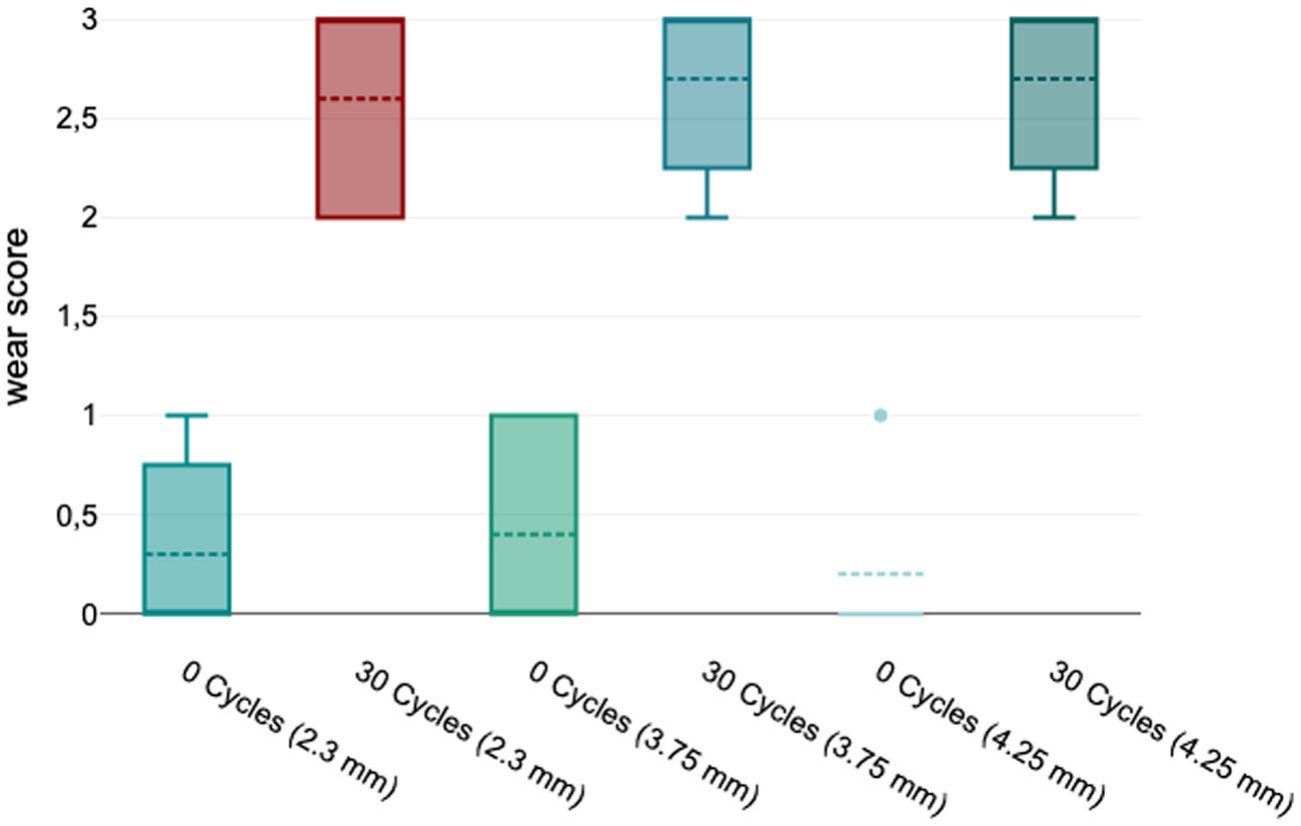
Boxplot representation of wear scores for zirconium dioxide drills with diameters of 2.3, 3.75, and 4.25 mm before (0 cycles) and after 30 drilling cycles. The wear scores range from 0 (no visible wear) to 3 (significant wear with chipping). For all diameters, the wear scores increased significantly after 30 cycles, as indicated by the higher medians and reduced variability in the 30‐cycle groups. The 0‐cycle groups showed minimal wear, with median values close to 0. The dashed lines represent the median wear score, whereas the boxes and whiskers illustrate the interquartile range (IQR) and data variability. Outliers are marked as individual points.

**Figure 6:**
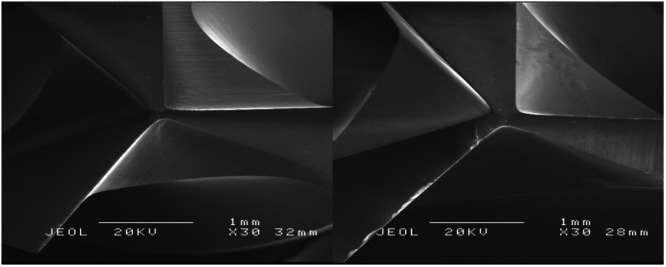
SEM images of a zirconia implant drill before and after repeated use and sterilization. The left image shows the cutting edge of a zirconia implant drill before repeated use and sterilization, demonstrating a well‐preserved cutting surface. The right image illustrates the wear and visible cracks and surface degradation of the same drill after 30 sterilization cycles and use, highlighting visible signs of material damage such as chipping and edge degradation. Both images were captured under a scanning electron microscope (SEM) at a magnification of 30x, with an accelerating voltage of 20 kV.

**Figure 7:**
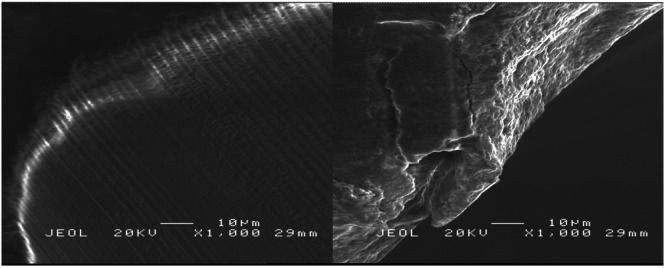
SEM images of a zirconia implant drill before and after repeated use and sterilization. The left image shows the cutting edge of the zirconia drill before repeated use and sterilization, illustrating a well‐maintained surface with no signs of wear. The right image depicts the drill after 30 sterilization cycles and use, with visible cracks, surface degradation, and chipping along the cutting edge. Both images were captured under a scanning electron microscope (SEM) at a magnification of 1000x, with an accelerating voltage of 20 kV.

The wear assessment of zirconium dioxide drills with diameters of 2.3, 3.75, and 4.25 mm demonstrated a significant increase in wear grades following 30 drilling cycles, as detailed in Tables 1, –, 3 and Figure 5. Drills subjected to 30 cycles showed predominantly higher wear grades (2–3), indicating substantial wear and deformation, whereas the unused drills (0 cycles) were predominantly classified as Grade 0 or Grade 1, signifying minimal or no wear (Figures 6 and 7).

**Table 1:** Wear assessment of zirconium dioxide drills with diameters of 2.3, 3.75, and 4.25 mm before use (0 cycles) and after 30 drilling cycles. Wear grades are categorized as follows: Grade 0 indicates no visible wear or deformation, Grade 1 reflects minor wear with intact cutting edges, and Grade 2 denotes significant wear, including chipped cutting edges.

0 Cycles (2.3 mm)	30 Cycles (2.3 mm)	0 Cycles (3.75 mm)	30 Cycles (3.75 mm)	0 Cycles (4.25 mm)	30 Cycles (4.25 mm)
0	3	1	2	0	3
0	2	0	3	1	3
0	3	0	2	0	3
0	3	0	3	0	2
1	2	0	3	0	3
0	3	1	3	0	2
1	2	0	3	0	3
0	2	1	2	0	3
1	3	0	3	1	2
0	3	1	3	0	3

**Table 2:** Statistical summary of wear grades for zirconium dioxide drills with diameters of 2.3, 3.75, and 4.25 mm, evaluated before use (0 cycles) and after 30 drilling cycles. Key metrics include the number of drills assessed, the average wear grade, the middle value of the wear grades, and variability in wear grades within the group.

	n	Mean	Median	Standard deviation
0 Cycles − 2.30	10	0.3	0	0.48
30 Cycles − 2.30	10	2.6	3	0.52
0 Cycles − 3.75	10	0.4	0	0.52
30 Cycles − 3.75	10	2.7	3	0.48
0 Cycles − 4.25	10	0.2	0	0.42
30 Cycles − 4.25	10	2.7	3	0.48

**Table 3:** Statistical analysis of drill wear for three diameters: 2.30 mm, 3.75 mm, and 4.25 mm. For each diameter, the Mann–Whitney U test results are reported, including the test statistic (U), the standardized z‐score (z), and p‐values from both asymptotic and exact calculations. The effect size (r) quantifies the strength of the differences observed. All comparisons show significant differences (*p* < 0.001) with effect sizes (r ≥ 0.88).

Drill diameter (mm)	U	z	Asymptotic *p*	Exact *p*	*r*
2.30	0	−3.94	< 0.001	< 0.001	0.88
3.75	0	−3.94	< 0.001	< 0.001	0.88
4.25	0	−4	< 0.001	< 0.001	0.9

Quantitative analysis revealed a significant increase in the mean wear grades across all diameters. For drills with a diameter of 2.3 mm, the mean wear grade increased from 0.3 (0 cycles) to 2.6 (30 cycles). Similar trends were observed for 3.75 mm drills (0.4–2.7) and 4.25 mm drills (0.2–2.7). Median wear grades also increased consistently from 0 to 3 across all diameters, indicating uniform progression in wear with repeated use. The low standard deviations in the 30‐cycle group underscore the reproducibility of the observed wear patterns.

Statistical analysis using the Mann–Whitney U test confirmed significant differences in wear grades between the control and test groups for all diameters (*p* < 0.001). The effect sizes (*r* = 0.88–0.90) indicate a robust association between the number of cycles and the extent of wear.

## 5 Discussion

In this experimental study, zirconia implant drills of varying diameters (2.3, 3.75, and 4.25 mm) were used to prepare implant sites in porcine mandibular bone. Wear on the main cutting edge was assessed using scanning electron microscopy (SEM) at 1000x magnification and graded according to a defined scoring system. The results revealed a statistically significant increase in wear after 30 drilling cycles across all diameters (*p* < 0.001), with a strong effect size (*r* ≥ 0.88). This finding highlights the rapid wear progression of zirconia drills, particularly after repeated use.

Reusable implant drills are designed to endure approximately 25 osteotomies, given that 2.5 implants are placed per surgical procedure on average (Elani et al. 2018). However, the study showed substantial wear after only 30 cycles, indicating potential limitations in the durability of zirconia drills under simulated clinical conditions. In contrast, studies on medical steel drills have reported significantly less wear after similar or greater usage. For example, one study found no significant wear after 30 drilling and sterilization cycles, with notable changes occurring only after 60 cycles (Alevizakos et al. 2023). Similarly, Koo et al. observed that pilot drills, which penetrate cortical bone, experience greater wear compared to depth drills, recommending their more frequent replacement (Chakraborty et al. 2024). Here, the results corroborate these findings, as the primary cutting edges of all zirconia drills showed clear wear and deformation after 30 cycles.

Increased wear of zirconia drills has significant clinical implications. As the drill wears, cutting efficiency declines, leading to higher heat generation, which can damage bone tissue and impair osseointegration (Chakraborty et al. 2024). Additionally, worn drills may lose precision, potentially affecting implant placement accuracy and compromising long‐term outcomes (Takács et al. 2023). Regular monitoring and timely replacement of drills are crucial to ensure optimal surgical performance and minimize complications (Hussain et al. 2023). Future research should focus on the link between drill wear and clinical outcomes to better understand its impact.

The differences in cutting performance and wear between zirconia and stainless‐steel drills can be attributed to material properties. Zirconia drills have smoother surfaces, as demonstrated by Batista Mendes et al. but their brittleness may predispose them to chipping under repeated mechanical stress (Batista Mendes et al. 2014; Augusto Alves Bento et al. 2024). In contrast, stainless‐steel drills display more pronounced signs of wear but maintain functional integrity over a longer usage period. This brittleness, compounded by the need for zirconia drills to endure cortical bone penetration, likely accelerates their wear.

The porcine mandibular bone used in this study provided a suitable substrate for simulating human jaw anatomy, as it closely replicates the cortical and cancellous bone structure found in humans (Tsiagadigui et al. 2022; Soldatos et al. 2022). However, natural variability in bone density between regions (D1‐D4) may have influenced wear outcomes, as cortical bone generates greater resistance and heat compared to cancellous bone. Synthetic jaw models, cadaveric specimens, and advanced 3D‐printed models have been suggested as alternatives for simulating clinical conditions (Llopis‐Grimalt et al. 2024; Fleps et al. 2020; Msallem et al. 2024). Although synthetic models offer standardization, differences in mechanical properties compared to natural bone can alter drill performance and wear (Chakravarthy et al. 2022). Future studies comparing these models with natural substrates are warranted.

Drilling parameters were standardized to ensure reproducibility. An axial pressure of 20 N, determined through studies by experienced clinicians, was applied consistently. Although the literature reports varying recommendations for axial pressure, ranging from low contact pressure to values as high as 24 N (Chen et al. 2024; Brisman 1996; Rugova and Abboud 2024), maintaining a constant force ensured controlled experimental conditions. The drilling speed was set at 800 rpm, in line with manufacturer guidelines and widely accepted clinical recommendations (Bernabeu‐Mira et al. 2021; Delgado‐Ruiz et al. 2018).

Sterilization and reprocessing of the drills may have contributed to the observed wear. Each sterilization cycle subjects the drills to thermal, pressure‐related, and mechanical stress, which can accelerate deterioration of the cutting edges (Alevizakos et al. 2021). Although single‐use drills eliminate this concern, their high cost and low infection risk in controlled settings justifies the continued use of sterilizable drills in clinical practice (Badrfam 2021). Zirconia, as demonstrated in previous studies, has excellent sterilizability and mechanical strength (Koo et al. 2015). For instance, Maccauro et al. reported fracture resistance values of up to 1484.89 N for zirconia, underscoring its mechanical reliability for dental applications (Bai et al. 2022; Maccauro et al. 2009). However, low‐temperature degradation (LTD) of zirconia, a well‐documented phenomenon during autoclaving, could potentially influence the findings of this study. LTD occurs when zirconia undergoes a phase transformation from its metastable tetragonal phase to the monoclinic phase at lower temperatures, which can lead to surface roughening and a reduction in mechanical properties (Lucas et al. 2015). Although the drills in the present study were sterilized according to standard protocols, it is possible that some degree of LTD occurred, contributing to the wear observed. However, the zirconia drills used in this study were made from Yttria‐stabilized zirconia (Y‐TZP), a material known for its enhanced resistance to LTD and greater fracture toughness compared to other zirconia variants (Arellano Moncayo et al. 2023). This choice of material was made to ensure greater durability and performance. Although LTD may have had a minor effect on the material's surface integrity, the use of Y‐TZP likely minimized its impact. Future research that explores the relationship between LTD and drill wear could provide more insights into its role in the clinical performance of zirconia drills.

Despite its promising mechanical properties, zirconia drills face economic challenges due to high manufacturing costs. Current production relies on milling, which is expensive and time‐consuming. Innovations such as injection molding for other ceramics like lithium disilicate and advancements in 3D printing technology could significantly reduce production costs. Other manufacturers already offer 3D‐printed Y‐TZP components, paving the way for cost‐effective, customizable zirconia drill production (Kongkiatkamon et al. 2023; Zenthöfer et al. 2024). However, the potential for material abrasion and contamination during osteotomy preparation remains a concern that requires further investigation.

In addition to the inherent material properties of zirconia, factors such as implant site preparation and bite forces significantly influence drill performance and wear. Effective site preparation is crucial for minimizing heat generation during drilling, which can otherwise impair osseointegration and bone healing. The present study supports the notion that zirconia drills, like stainless‐steel drills, perform well under standardized conditions; however, the results also emphasize the importance of drilling parameters and bone characteristics. The study by Lin et al. provides further evidence, showing that zirconia drills generate notably lower temperatures (32.98 ± 1.21°C) compared to stainless‐steel drills (45.48 ± 1.31°C) (Lin et al. 2024). This thermal advantage reduces the risk of thermal damage to bone, which is critical for maintaining bone vitality and successful implant integration. Moreover, although the present study did not directly assess bite forces, they are likely to play a key role in both drill wear and cutting efficiency. Variations in bite forces during drilling could impact the longevity of zirconia drills, as they are subjected to different stresses under real clinical conditions. Further studies are needed to examine how bite forces, in combination with other clinical factors, affect drill wear and thermal performance in vivo, as this will provide valuable insights into the clinical durability of zirconia drills.

This study has limitations that must be acknowledged. First, the use of porcine bone, although a reliable model, cannot fully replicate the complexity and variability of human bone. Second, the study evaluated wear under controlled experimental conditions, which may differ from clinical scenarios involving variable pressures, angles, and patient‐specific factors. Additionally, the sample size was limited to a predefined number of cycles and drill diameters, warranting further studies with larger sample sizes and extended usage cycles.

## 6 Conclusion

In conclusion, zirconia drills show significant wear after 30 drilling cycles, particularly at the primary cutting edge, likely due to their mechanical properties and interaction with cortical bone. Although zirconia represents a promising alternative to stainless steel for implant drills, improvements in manufacturing processes and further investigation into their clinical durability and cost‐effectiveness are essential.

## Author Contributions

V.A. conceptualized the study, supervised the experimental design, performed statistical analyses, and drafted the manuscript. R.M. contributed to the study design, was responsible for data acquisition, carried out the initial data analysis, and reviewed the manuscript critically for important intellectual content. A.C.P. assisted in the experimental setup and conducted laboratory work. C.v.S. provided technical expertise, contributed to the interpretation of results, and participated in the critical revision of the manuscript. All authors have read and approved the final manuscript.

## Ethics Statement

The authors have nothing to report.

## Consent

The authors have nothing to report.

## Conflicts of Interest

The authors declare no conflicts of interest.

## Data Availability

The data that support the findings of this study are available from the corresponding author upon reasonable request.
