# Correction to “Effects of Repeated Use and Sterilization on the Wear of Zirconia Implant Drills: A SEM‐Based Analysis”

**DOI:** 10.1002/cre2.70157

**Published:** 2025-06-11

Alevizakos, V., Mosch, R., Platte, A.‐C., and von See, C. 2025. “Effects of Repeated Use and Sterilization on the Wear of Zirconia Implant Drills: A SEM‐Based Analysis.” *Clinical and Experimental Dental Research* 11: e70088. https://doi.org/10.1002/cre2.70088

Kemo Funder Statement was missing in the article. This statement has now been included.

We apologize for this error.

